# Experimental Research on the Ecological Recovery of Metals from Used Ni-MH Batteries

**DOI:** 10.3390/ma18245549

**Published:** 2025-12-10

**Authors:** Valeriu Gabriel Ghica, Florin Miculescu, Ana Vasile, Narcis Daniel Saftere, Angelos P. Markopoulos, Șener Karabulut, Mircea Ionuț Petrescu, Eugenia Tanasă, Anca Icleanu

**Affiliations:** 1 Department of Engineering and Management of Metallic Materials Production, National University of Science and Technology Politehnica Bucharest, 313 Splaiul Independentei, 060042 Bucharest, Romania; gvghica@yahoo.com (V.G.G.); anavasile01@yahoo.com (A.V.); narcistoko@yahoo.com (N.D.S.); ipetrescu@yahoo.com (M.I.P.); vanca_rhl@yahoo.com (A.I.); 2 Department of Metallic Materials Science, Physical Metallurgy, National University of Science and Technology Politehnica Bucharest, 313 Splaiul Independentei, 060042 Bucharest, Romania; f_miculescu@yahoo.com; 3 Manufacturing Technology Laboratory, School of Mechanical Engineering, National Technical University of Athens, 9 Iroon Polytechniou, 15772 Zografou, Greece; 4 Department of Mechanical Program, Hacettepe University, 06930 Ankara, Turkey; senerkarabulut@hacettepe.edu.tr; 5 Department of Physics, National University of Science and Technology Politehnica Bucharest, 313 Splaiul Independentei, 060042 Bucharest, Romania; eugenia.vasile@upb.ro

**Keywords:** batteries, recycling, rare earths, metal recovery

## Abstract

The presented research is focused on identifying a cheap and environmentally friendly solution for recovering useful non-ferrous metals contained in used Ni-MH batteries—more specifically, in batteries that power medical equipment, i.e., portable defibrillators. The cathodic paste of Ni-MH batteries contains Ni(OH)_2_ as an active material to which Zn, Co and Mn can be added. The paste is impregnated into a support mesh made of nickel. The anodic paste of Ni-MH batteries contains mixtures of rare earths capable of storing the released hydrogen. The paste is mixed with a binder and pressed onto a metal grid made of nickel alloy. After manual disassembly, the components of the Ni-MH batteries were analyzed by X-ray Fluorescence Spectroscopy (XRF) before and after the separation/recovery operation. To separate the cathode and anode paste from the metal supports (grids, metal meshes), an ultrasonic bath with appropriate solutions was used, and the optimal working parameters were established. The recovery of the anode paste was achieved by completely passing the rare earths into the citric acid solution used for ultrasonication; the nickel mesh was cleaned of the Ni(OH)_2_ paste using water as the ultrasonication medium. After separation from the metal supports, the anode and cathode pastes were analyzed and characterized by XRF, optical and electron microscopy (SEM, EDX). The results obtained are of real interest for those who study the recycling of Ni-MH batteries; the use of ultrasound in a low-concentration citric acid environment for the purpose of recovering rare earths can be an economic and ecological alternative for battery recycling.

## 1. Introduction

Batteries are an indispensable daily presence for 21st-century man; life cannot be imagined today without a phone, laptop and a multitude of other portable devices and tools powered by batteries. Disposable or rechargeable batteries, of a wide variety (lead–acid, Ni-MH, Li-ion), are the focus of researchers’ attention in order to improve their performance and to find economic and ecological ways of recycling them to valorize the useful elements they contain. The most performant batteries, intensively marketed, are those based on lead–acid, Ni-MH and Li-ion systems [1,2,3]. The performances of these three types of batteries are remarkable (Table 1).

Lead–acid batteries, although not very energy-dense, can provide very high currents at low cost, making them suitable for starting internal combustion vehicles and as backup power in telecommunications. These batteries perform well in extreme temperatures and are inexpensive. Recycling of these batteries is known and functional, although it still needs improvement. Ni/MH batteries, which replaced the (toxic) Ni/Cd ones, are in fierce competition with Li-ion batteries; initially, they were much more expensive, but, after 2010, they became cheaper by reducing the content of expensive and scarce metals (Co, Ni) in their composition.

Today, these two types of batteries share the market: Li-ion batteries have captured the market for small devices (phones, cameras), and electric vehicle (e.g., LiFePO_4_) batteries, which also offer the safest chemical configuration; Ni/MH batteries dominate the AA battery sector (producing voltages of 1.3 V compared to 3.2–3.8 V in the case of Li-ion batteries) and applications that require high-current discharge rates (defibrillators). It is estimated that by 2030 Li-ion batteries will cover 95% of the market. Today, we are going through a period of intense research aimed at valorizing the non-ferrous metals contained in used batteries (Li-ion, Ni-MH), which, due to their high content of Ni, Co, Mn, Zn and rare earths—which are useful, expensive, scarce and often difficult to access—are transformed from waste into true secondary sources of raw materials [4,5,6,7,8,9]. Following the research undertaken in recent years, used batteries have become suppliers of materials used to obtain colored glass, magnetic materials, graphene, electrical insulators, raw materials for electroplating processes, etc.

Starting in the 1980s and especially after 1990, the introduction of rare earths in Ni-MH (Nickel–Metal Hydride) batteries began, with the discovery that La, Ce, Pr and Nd—most often in the form of a mixture called mischmetal—presented superior characteristics for reversible storage of hydrogen in the anode of batteries, eliminating from the market Ni-Cd batteries that contained toxic Cd. Rare earths can absorb and then release a greater amount of hydrogen, thus resulting in a higher energy density of the battery. RE allow the acceleration of the hydrogen absorption/release processes, which leads to higher charging/discharging speeds; in addition, they extend the battery life through increased stability and resistance to degradation [10,11].

As a rule, on the labels and packaging of Ni-MH batteries, as well as Li-ion batteries, one can find information about the capacity/voltage of the batteries, information related to operational safety and information about the lifespan of the batteries; however, we do not find any information regarding the composition of the materials/metals present in the anodic/cathode paste, in the anodic/cathode support grids and electrolyte, regarding the possibilities of recovering and reusing the valuable materials found in these batteries that have reached the end of their service life. The presence of Ni, Co and rare earths in these discarded batteries transforms used NI-MH batteries from waste into rich secondary sources of raw materials.

The exact recipes (chemical composition, structure) of the metals and alloys found in the batteries are considered a trade secret, either to protect companies from competition or to avoid concerns caused to users who could thus find out about the ecological and social impact produced by the extraction and separation of rare earths, nickel and cobalt from ore.

Today, rare earths have become an indispensable component of modern technologies, namely communications, medical imaging equipment, production of permanent magnets, energy storage elements, lasers, etc.; therefore, the recovery of these elements from used batteries is a major concern of research centers. Table 2 shows an example of the information provided in the product data sheet by a well-known battery manufacturer (Panasonic, Osaka, Japan).

It can be seen that in the product sheet the information is incomplete, which denotes legislative gaps and ambiguous regulations; the data provided is able to discourage potential entrepreneurs willing to try recycling such batteries. Only on 28 June 2023 did the Council of the European Parliament adopt a new legislative act—Regulation on Batteries and Waste Batteries 2020/0353, amending Directive 2008/98/EC and Regulation (EU) 2019/1020 and repealing Directive 2006/66/EC—which strengthens sustainability rules for batteries and waste batteries. This regulatory act stipulates, among many other measures, that batteries will have to contain documentation for recycled content. It also introduces labeling and information requirements on battery components, recycled content, and a battery passport with a QR code. Moreover, the regulation establishes that by 2027, batteries incorporated in appliances should be able to be dismantled and replaced by the end user.

## 2. Materials and Methods

### 2.1. Materials

The authors focused their attention on the recovery of useful metals contained in used Ni-MH batteries. Recycling of used Li-ion and Ni-MH batteries is mainly carried out in Asia (China, Japan, Republic of Korea), which are the main producers worldwide; the companies operating in these countries have access to detailed information regarding the chemical composition of the batteries, information that is considered confidential. No other company allows itself to recycle batteries whose composition is unknown. Therefore, recycling centers belong to the companies producing such components. Only starting in 2025 has the EU imposed the requirement to indicate the chemical composition on batteries sold within the EU. Consequently, the data published in the specialized literature on this topic refer mainly to research-level studies. Regarding Ni-MH batteries, pyrometallurgical processes for recovering rare earths start from the fact that rare earths and transition metals present in these batteries (Fe, Co, Ni) have different affinities toward oxygen. Through melting with the addition of fluxes (CaO/CaF_2_/SiO_2_), an Fe–Ni–Co alloy is obtained along with a slag that concentrates the rare earths in the form of oxides (SiO_2_CaOReO). Separation of rare earths from the slag requires concentrated sulfuric and hydrochloric acid solutions, which are very aggressive to the environment. Hydrometallurgical technologies for recycling Ni-MH batteries are based on chemical dissolution, precipitation and purification processes to recover Ni and Co [13,14,15,16]. After dismantling and separating the components, the used batteries are subjected to crushing and grinding operations. The ground cathodes and anodes are dissolved in acidic (sulfuric acid, hydrochloric acid) or alkaline (caustic soda) solutions to solubilize Ni and Co. Precipitating agents are then added to the dissolved metal solutions to separate them as insoluble compounds; Ni and Co can be precipitated with compounds that form insoluble salts (hydroxides, oxides or carbonates) in acidic solutions or with bases (NH_4_OH) in alkaline solutions. Both variants are expensive and polluting. The proposed option is fast, non-polluting and cheap.

**Figure 1:**
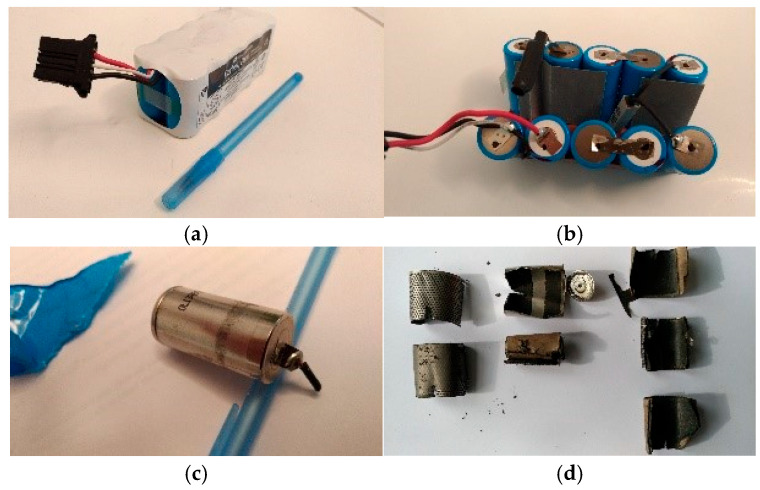
Manual disassembly of used Ni-MH batteries. (**a**) Original battery for defibrillator. (**b**) The defibrillator battery consists of 10 Ni-MH batteries; (**c**) Ni-MH battery before disassembly; (**d**) Ni-MH battery disassembled and separated into components.

The Ni-MH batteries considered are rechargeable medical batteries used in portable defibrillators (Figure 1a). In this study, one original battery for defibrillator spent Ni-MH batteries composed of 10 Ni-MH elementary batteries was manually dismantled and their components separated (Figure 1b–d). The novelty of the research lies in developing fast, low-cost and reagent-efficient ultrasonication-based methods for the selective separation and recovery of both cathode and anode active pastes, without the need for complex chemical treatments. For the cathode, we investigated the detachment of the Ni(OH)-containing paste from the nickel mesh using ultrasonication in water. The recovery efficiency was evaluated as a function of ultrasonication time and bath power, while simultaneously monitoring the influence of bath power on temperature. The cathode before treatment, the cleaned support grid and the recovered paste were subsequently analyzed and characterized. The study further advances the state of the art by applying two complementary ultrasonication-based approaches for the recovery of the anode paste containing rare earth elements. Specifically, (i) ultrasonication in water was used to detach and recover the paste (containing Ce, La, Y) from the nickel–alloy support grid, and (ii) ultrasonication in 1 M citric acid enabled controlled dissolution of the anode paste and the transfer of rare earths into solution. The efficiency of both processes was strongly affected by bath power and temperature. The anode before treatment, the cleaned support grid and the recovered anode paste were also thoroughly analyzed and characterized. These results demonstrate a simple, scalable and environmentally benign route for the recovery of valuable metals from spent Ni-MH batteries.

### 2.2. Laboratory Equipment Used and Working Method

Sections were cut from the anode and cathode, respectively, and weighed before and after cleaning in order to calculate the cleaning efficiency. Cleaning efficiency was calculated with the following equation:

(1) $$\eta =\frac{m_{i}-m_{f}}{m_{i}}\times 100$$

where

*η*—cleaning efficiency;
*m_i_*—initial weight of anode (cathode) piece, g;
*m_f_*—final weight of anode (cathode) piece, g.

The samples were weighed with an electronic balance type EMB 200-3, Kern, (max. weighing weight: 200 g; reproducibility = 0.001 g). The operation of separating the support paste was carried out in an Emmi Emmi-12HC ultrasonic bath. The characteristics of the ultrasonic bath are as follows: the maximum volume of the stainless-steel tank is 1.2 L; the tank has the following dimensions: 200 × 100 × 65 mm; cleaning frequency = 45 kHz; cleaning time = 1–60 min; the bath temperature can vary between 20 and 80 °C; maximum ultrasonic power = 100 W, ultrasonic power can be set to one of three levels: 50/75/100 W. The water temperature was measured continuously. Water and a solution of about 1 M citric acid were used as a medium.

The research was undertaken within the Hydrometallurgy Laboratory of the Department of Engineering and Management of Metallic Materials at POLITEHNICA University Bucharest, which has expertise in the field of recycling lead–acid and Li-ion batteries [17,18,19]. X-ray diffraction analysis was carried out using the D8-Discover diffractometer, Bruker, Ettlingen, Germany, with Cu primary radiation (λ = 1.540598 Å), parallel geometry and a 1D LynxEye detector (Bruker, Germany) on the secondary side. The diffractograms were obtained with an angular increment of 0.04°, at a scanning speed of 1 s/step, in the Physical-Chemical Testing Laboratory, LI-MAT, within the National Research and Development Institute for Electrical Engineering ICPE-CA, Bucharest. The identification of the crystallographic phases was carried out using the ICDD PDF 2 Release 2022 database.

Samples from the cathode and anode of Ni-MH batteries were analyzed by scanning electron microscopy using a Quanta Inspect F50 (FEI Company, Eindhoven, The Netherlands) with a field emission gun (FEG) (Thermo Fisher Scientific, Waltham, MA, USA) with 1.2 nm resolution, and an energy dispersive X-ray spectrometer (EDX) (Thermo Fisher Scientific, Waltham, MA, USA) with a resolution of 133 eV at MnKα. A Thermo Fisher Quattro S electron microscope (Thermo Fisher Scientific, Waltham, MA, USA) was also used.

## 3. Results

### 3.1. Separation/Recovery of Cathode Paste (Ni(OH)_2_) from the Support Mesh (Made of Ni)

**Figure 2:**
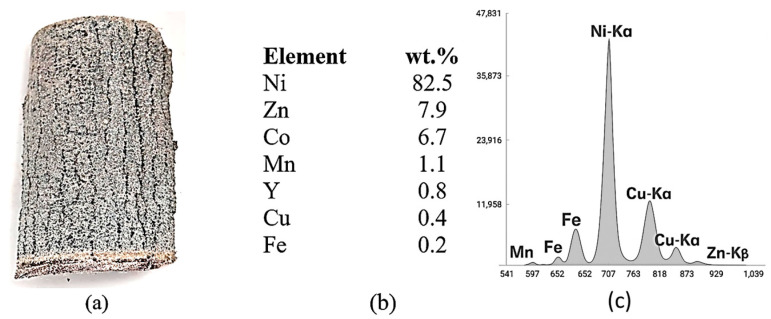
X-ray fluorescence analysis on a cathode section before ultrasonication: (**a**) the aspect of the cathode of the Ni-MH battery; (**b**) XRF quantitative analysis to whole cathode (mesh/screen metal + paste); and (**c**) XRF spectra corresponding to whole cathode analysis.

**Figure 3:**
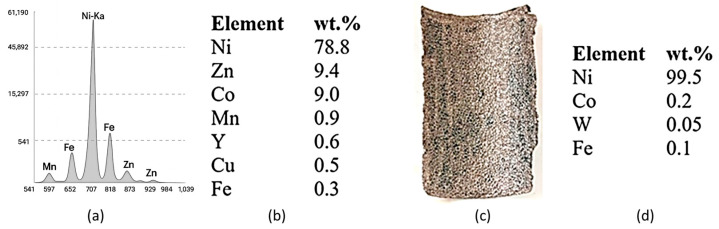
X-ray fluorescence analysis on a cathodic section after ultrasonication: (**a**) cathodic paste XRF spectra analysis; (**b**) XRF quantitative analysis results; (**c**) the aspect of the of mesh/screen metallic support of cathode; (**d**) XRF quantitative analysis results of the mesh/screen metallic support of cathode.

Sections of the cathode of the used Ni-MH battery were analyzed by X-ray fluorescence before (Figure 2) and after the ultrasonication operation in water (Figure 3).

**Figure 4:**
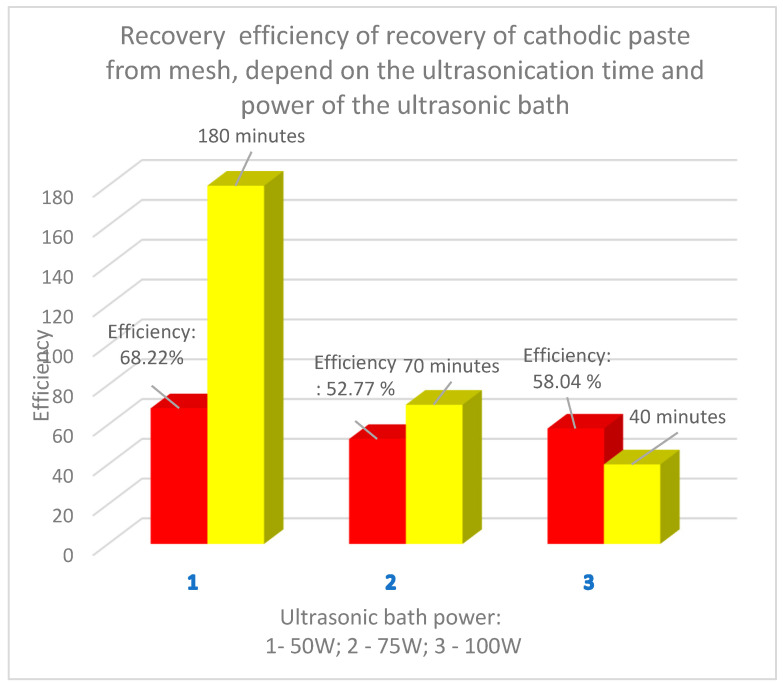
Cleaning efficiency depending on time and power of the ultrasonic bath.

XRF analyses performed on sections cut from the cathode before ultrasonic cleaning revealed the presence of Ni, Zn, Co, Mn, Y, Cu and Fe. After ultrasonic cleaning in water, the cathodic paste was detached from the support metal mesh made of Ni. Following XRF analysis, the following elements were identified in the recovered paste (washed and dried): Ni, Zn, Co and traces of Cu and Fe. The evolution of the cleaning efficiency as a function of time and ultrasonic bath power is presented in Figure 4.

The values recorded in Figure 4 are the average values obtained from three experiments performed for each power level.

**Figure 5:**
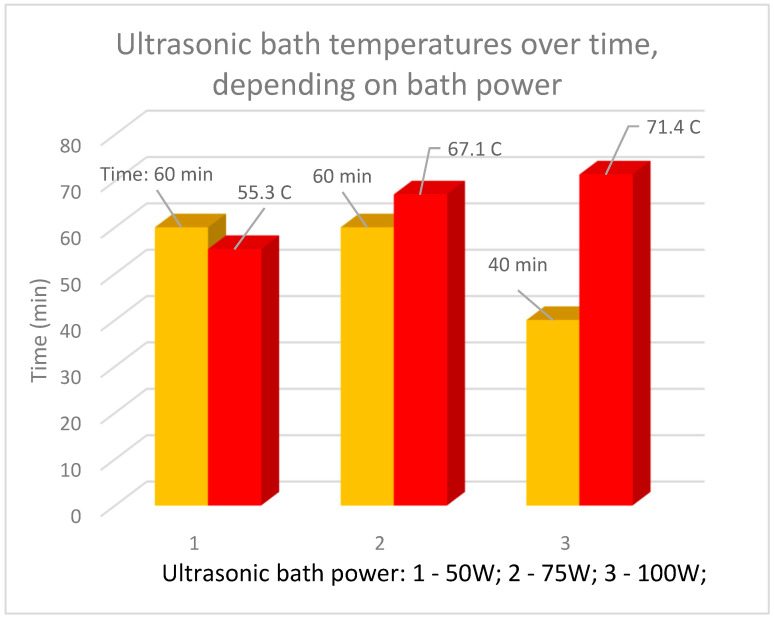
Increase in ultrasonic bath temperature over time, depending on bath power.

Figure 5 graphically shows the increase in water temperature in the ultrasonic bath, depending on the bath power.

The values recorded in Figure 5 are the average values obtained from three experiments performed for each power level.

From Figure 5 it can be seen that a high temperature of the ultrasonic bath (from 55.3 to 71.4 °C) is reached much faster if maximum bath power is employed (from 50 W to 100 W). The experiments were carried out starting from the temperature of the bed (20 °C). Taking into account the conclusions drawn from the previous experiments, it was decided to perform the ultrasonication operation in water of the cathode of Ni-MH batteries by preheating the bath to 40 °C. Rapid separations/cleanings (15 min) of the paste with a majority content of Ni(OH)*_2_* from the support mesh made of Ni were obtained.

### 3.2. Characterization of the Cathode Support Mesh After Ultrasonication

**Figure 6:**
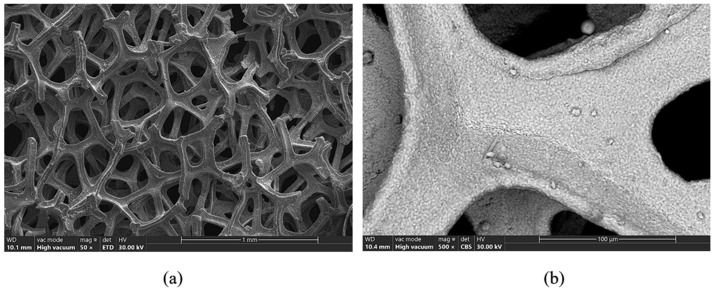
(**a**) Structure of the cathode support mesh, (**b**) cathode support mesh lamella.

From Figure 6a, it is observed that the support mesh presents a porous structure with open and interconnected pores with complex irregular shapes (eyes). The width of the lamellae that form the meshes of the network is between about 40 to 80 µm (Figure 6b). The morphology of the lamella surface presents irregularities of the order of µm.

**Figure 7:**
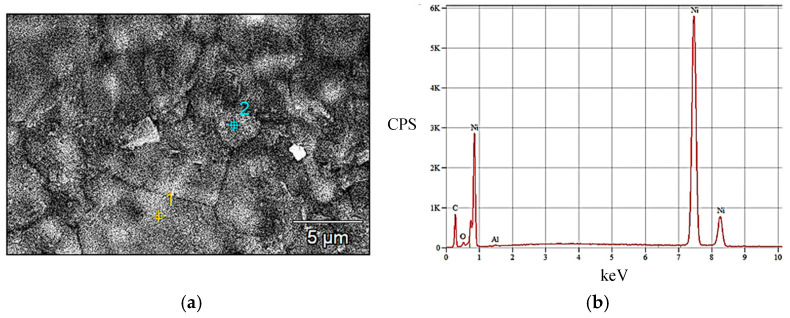
EDS analysis performed on the cathode support mesh. (**a**) Cathode support mesh (SEI image); (**b**) EDX compositional analysis.

The compositional analysis performed by the EDS method was performed at 5 points on each sample, the quantitative result presented being their average. Figure 7a captures in a secondary electron microscopy image an area of the cathode support mesh in which an EDX compositional analysis was performed (Figure 7b). It can be concluded that the cathode support mesh is made of nickel.

### 3.3. Characterization of Recovered Cathode Paste After Ultrasonication

**Figure 8:**
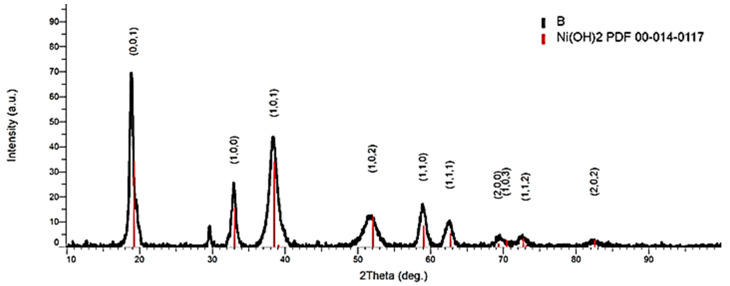
X-ray investigations on cathodic paste [19].

The cathode paste pressed on the nickel screen is made up of nickel hydroxide. The result of X-ray investigations on cathodic paste (Figure 8) was obtained and presented in a previous paper. With the “D” data tabulated in descending order and the corresponding relative intensities (Table 3), the compound Ni(OH)_2_ was identified.

**Figure 9:**
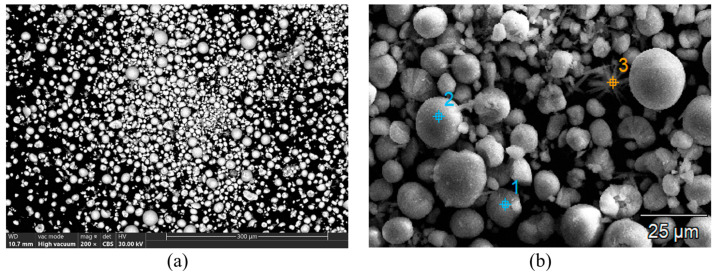
(**a**) The globular aspect of the particles obtained after ultrasonication of the cathode of used Ni-MH batteries; (**b**) the spots corresponding to the EDX analysis performed on globular particles obtained after ultrasonication of the cathode of used Ni-MH batteries.

**Figure 10:**
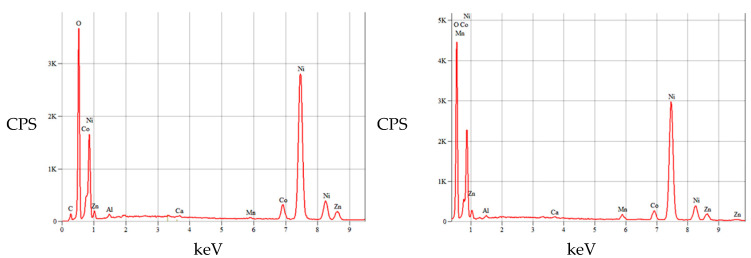
EDS spectra of two globular particles (X-ray emission spectrum); in addition to the well-defined presence of nickel, traces of other elements (Co, Zn, Mn) are also recorded.

The electron microscopy (EM) analysis performed on the cathodic powder resulting from ultrasonication reveals the particle morphology: predominantly globular particles with dimensions between 10 and 20 µm (Figure 9). The globular particles have approximately identical compositions with small variations in elemental concentrations (Figure 10).

There is a good reason to separate the cathode paste from the support mesh:

- If Ni is to be recovered and melted in an electric arc furnace, the cathode support mesh made of Ni, (T_melting_ = 1455 °C) together with the paste (Ni(OH)_2_), and Ni(OH)_2_ would decompose at 300–400 °C, releasing oxygen and transforming into NiO (temperature melting of NiO, T_melting_ = 1995 °C) which would make the nickel impure.
- Being separated from the cathode mesh, Ni(OH)_2_ could be used as a precursor for the NiO product: Ni(OH)_2_ heated at 400 °C for 1 h in air leads to its decomposition into NiO and water.

Ni(OH)_2_ → NiO + H_2_O

After that, NiO can be used for various electronic and electrochemical applications [20,21,22,23,24].

The recovered Ni(OH)_2_ is in the form of fine particles, offering a large contact surface area; these characteristics ensure high electrical conductivity and catalytic activity. The presence of traces of other elements (Co, Zn, Mn), which in this case are considered undesirable impurities, requires a purification step before reuse in the manufacture of electrodes or in catalytic applications.

### 3.4. Separation/Recovery of Rare-Earth-Containing Anode Paste from the Support Grid (Made of Ni Alloy)

**Figure 11:**
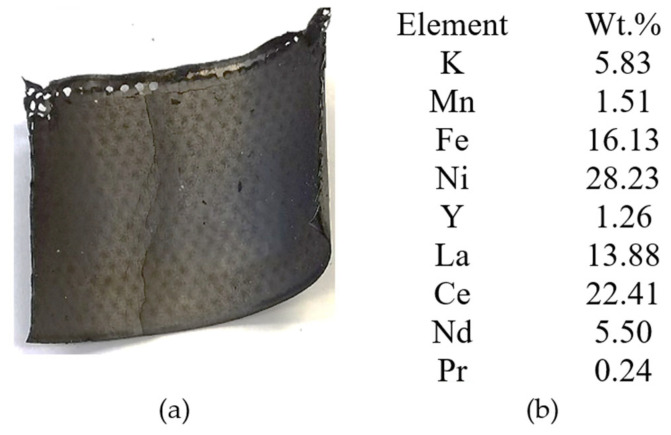
(**a**) Section of the anode grid, (**b**) Chemical analysis of the support metal grid.

The anode used in Ni-MH batteries consists of a support metal grid made of a Ni-Fe alloy on which an anode paste containing rare earths is compacted. The image of the analyzed section of the anode of used Ni-MH batteries before ultrasonication accompanied by the chemical composition (XRF analysis) are presented in Figure 11.

**Figure 12:**
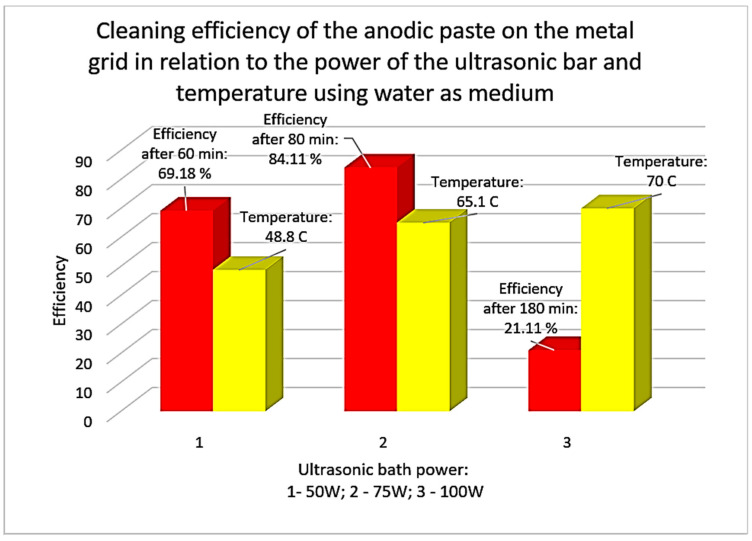
The cleaning efficiency of the anodic paste on the support grid resulting from ultrasonication in water correlated with the power of the ultrasonic bath and the temperature of the water in the ultrasonication tank.

As an ultrasonication medium for cleaning/separating the rare-earth-containing anode paste from the support grid, drinking water was used first and then a 1 M citric acid solution. The results of the research using water as the ultrasonic medium are presented in the diagram in Figure 12. Starting the research with water preheated to 40 °C, it can be seen that the efficiency varies between 21.11% and 84.11%. The maximum efficiency was reached after 80 min of ultrasonication at a bath power of 75 W. An increase in the ultrasonic power to 100 W decreases the efficiency and only has the effect of increasing the water temperature to 70 °C after 180 min.

The values recorded in Figure 12 are the average values obtained from three experiments performed for each power level.

**Figure 13:**
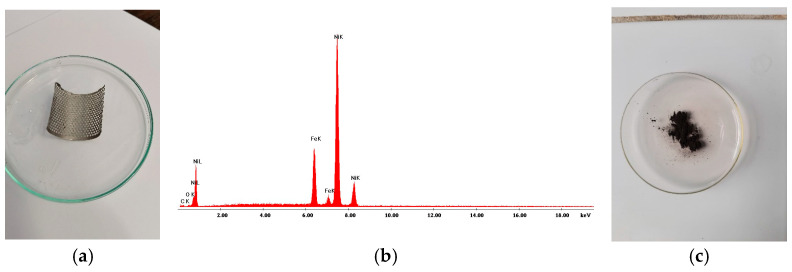
(**a**) Support grid after cleaning, (**b**) EDX analysis identifies the presence of Ni and Fe, (**c**) anodic paste recovered after ultrasonication in water.

**Figure 14:**
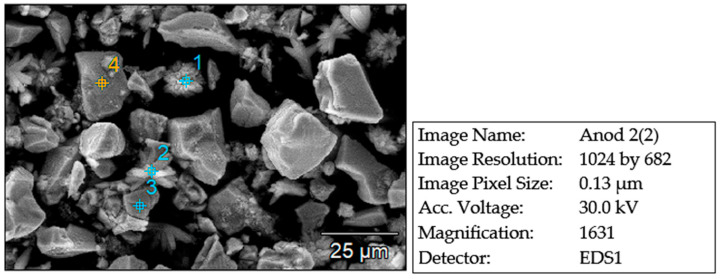
The morphology of the anodic paste recovered by ultrasonication in water.

**Figure 15:**
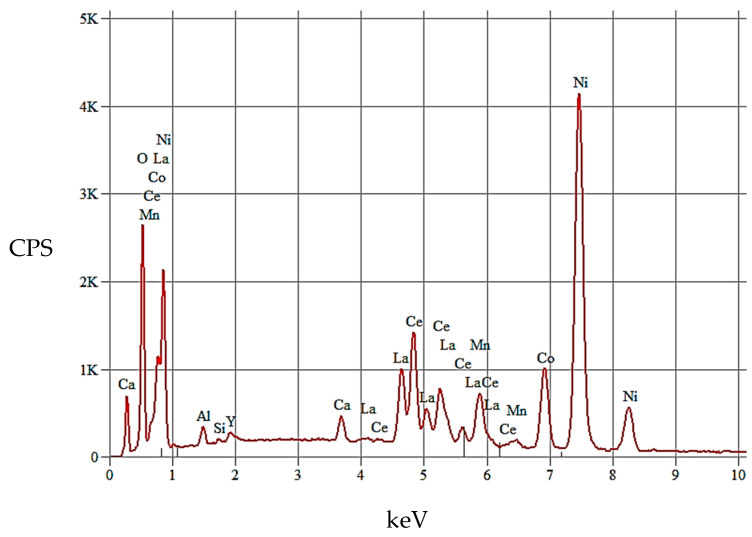
Chemical composition of the powder resulting from ultrasonication in water of a section of the anode of the rated Ni-MH battery (X-ray emission spectrum).

The ultrasonication time is high even in the case of achieving a high separation efficiency (over 80%). Next, the metal grid cleaned of the anodic paste (Figure 13a) was analyzed by EDX analysis (Figure 13b), which confirmed that it is made of a Ni-Fe alloy; the resulting paste (Figure 13c) was analyzed on the electron microscope (Figure 14 and Figure 15).

The morphology of the recovered anode paste is represented by agglomerations of angular particles with dimensions ranging from 5 to 25 µm, which were analyzed by EDX analysis, which highlighted the presence of rare earths (Ce, La, Y) along with Ni, Co, Mn, elements originating from the anode support grid.

### 3.5. Ultrasonication in Citric Acid Medium of an Anode Grid Section

**Figure 16:**
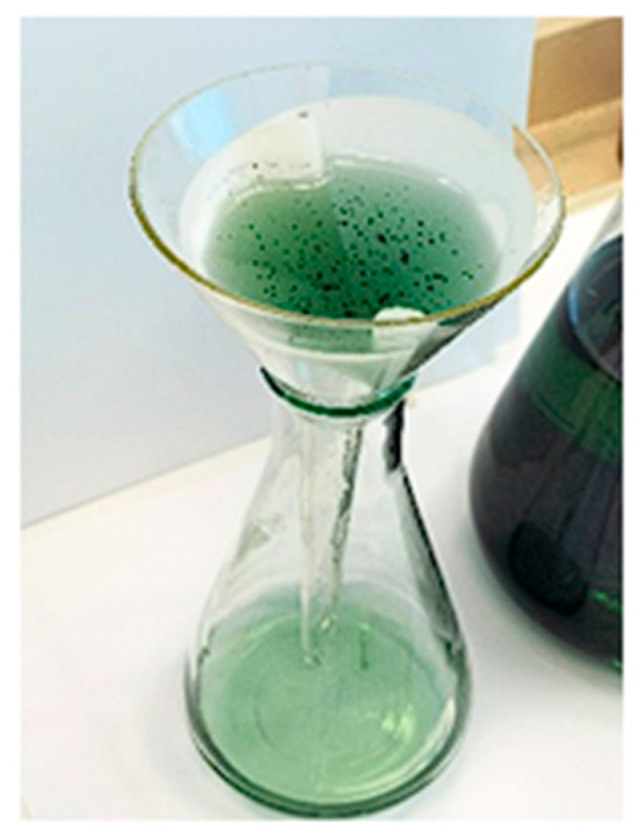
The resulting solution after ultrasonication in 1 M citric acid.

Using ultrasonication in a citric acid environment (about 1%M) (see Figure 16), the following was observed: if we start the cleaning process with a solution of about 1 M citric acid (C_6_H_8_O_7_) at room temperature (T-20 °C) and a maximum power of the ultrasonic bath (*p* = 100 W), the cleaning time of the anode grid is between 20 and 40 min.

A fresh citric acid solution was used for each set of ultrasonication tests; the used citric acid solution was not recycled. The cost of the solution is not significant (at least at this stage).

The weakly acidic solution (1 M citric acid) completely dissolves the anode paste (rare earths pass into solution). The result was expected; Brazilian [25] researchers proposed leaching the anode material solution with citric acid in order to obtain metallo-organic precursors that, when heated, lead to a mixture of nickel and rare earth oxides. The process is slow; however, dissolution occurs within 24 h under continuous stirring. The use of ultrasound accelerates the dissolution reaction, shortening the duration to minutes.

**Figure 17:**
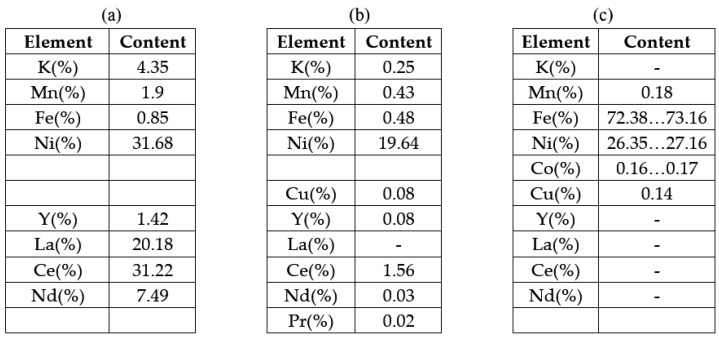
Results of XRF analysis performed on the following: (**a**) paste detached from the support grid, before ultrasonication in 1 M citric acid solution, (**b**) powder recovered after filtration of the aqueous solution ultrasonicated in 1 M citric acid solution, (**c**) metal support grid recovered after ultrasonication in 1 M citric acid solution.

As can be seen from Figure 17, the entire amount of Lanthanum and most of Ce and Nd went into the solution; traces of Nd remained and traces of Pr were also identified.

The research will continue with the analysis of the solutions resulting from the ultrasonication process in a weakly acidic environment; this will require specific equipment (Inductively Coupled Plasma Optical Emission Spectrometry—iCAP 6300 DUO, Thermo Fisher Scientific, Cambridge, UK), a specific method and ultrapure reagents.

The ultrasonication process causes cavitation phenomena generating high local temperatures and pressures, which accelerate the cleaning of the support and the dissolution of the paste. These processes are influenced by the oscillation frequency, the oscillation amplitude, the duration of the ultrasonication, the power of the source, etc. Research in this direction will continue in the next stages of the research, by using another laboratory installation—UP200Sta, 200 W power ultrasonicator for dispersion and sonochemical process (Hiescher Ultrasonics, Teltow, Germany).

## 4. Conclusions

After a manual separation into components (cathode, anode, electrical connections, casing) of Li-MH batteries used in medical applications, research focused on finding cheap and low-pollution technology for ultrasonic separation in water intended for human consumption, respectively, in a 1 M citric acid solution of the cathode/anode paste on the cathode screen/anode grid support in order to identify useful metals that can be recovered.

The cathode was ultrasonicated in a stainless-steel bath in drinking water; the support screen—made of Ni—was cleaned of the cathode paste. The cleaning efficiency slowly decreases from 68.22% to 58.04% as the ultrasonic bath power increases but with a significant reduction in the time required for cleaning (from 180 min to 40 min). If maximum bath power is used, namely 100 W, a high temperature of the ultrasonic bath, i.e., over 70 °C, is reached in short time.

Following electron microscopy analyses, it was found that the support mesh is made of nickel and has a porous structure with open and interconnected pores, complex irregular shapes and meshes with a lamella width of approximately 40 to 80 µm. Cathode paste, which is pressed on the nickel mesh, is made of globular particles of nickel hydroxide, particles with dimensions between 10 and 20 µm. The anode support grid is made of an Ni-Fe alloy. The recovery of the anode paste was performed by ultrasonication in drinking water and 1 M citric acid solution.

In the first case (drinking water as the ultrasonication medium), the maximum cleaning/recovery efficiency of the paste containing rare earths was 84.11%, a value reached after 80 min and at an ultrasonic bath power of 75 W. An increase in the purity of the ultrasonic bath does not help (recovery efficiency decreases) and increases the temperature to undesirable values. The recovered anode paste contains a mixture of rare earths (La, Ce) Yttrium and nickel detached from the support grid. The particle sizes resulting from ultrasonication in water are smaller than 25 microns.

In the case of performing the separation/recovery operation of the anode paste in 1 M citric acid solution, the rare earths passed into the solution in about 20 to 40 min, with the paste identifying elements detached from the support grid and traces of Ce.

Today, the search for ecological and cheap solutions for the recovery of useful metals contained in used Ni-MH batteries is of great diversity. Future research will continue in several directions:

- Finding a solution for mechanized dismantling and sorting of used Ni-MH batteries into components;
- Optimizing the process of recovering useful metals from used Ni-MH batteries by ultrasonication in a citric acid environment, because the process is cheap and environmentally friendly;
- Advantageous utilization of expensive and scarce non-ferrous metals and rare earths contained in used Ni-MH batteries.

## Figures and Tables

**Table 1:** Comparison between the performances of lead–acid, Ni/MH and Li-ion batteries.

	Lead–Acid	Ni/MH	Li-Ion
Voltage	2.1 V	1.3 V	3.8 V Volume energy
Weight energy	30–50 Wh/kg	70–80 Wh/kg (2001) 100–500 Wh/kg (2023)	120 Wh/kg (2001) 300 Wh/kg (2001)
Volume energy	80–90 Wh/dm^3^ (2001) 400 Wh/dm^3^ (2023)	150–200 Wh/dm^3^ (2001) 300 Wh/dm^3^ (2023)	150 Wh/dm^3^ (2001) 700 Wh/dm^3^ (2023)
Power	180 W/kg (2001) <1000 W/kg (2001)	200–300 Wh/kg (2021) 100–500 Wh/kg (2023)	500 Wh/kg (2021) 500–700 Wh/kg (2023)
Cycle life	<350	1000 (2001) 2000 (2023)	1500 (2001) 3000 (2023)
Cost	50–150 $/KWh	330 $/KWh (2001) 139 $/KWh (2023)	800 $/KWh (2001) 75–259 $/KWh (2023)

**Table 2:** Composition and ingredient information [12].

Common Chemical Name	CAS Number	Concentration/ Percentage Range
Nickel Hydroxide Cobalt Hydroxide	12054-48-7 21041-93-0	15–25% (15–30%) * 1–5%
Hydrogen-absorbing alloy	7440-02-0(Ni) 7440-48-4(Co) 7439-96-5(Mn) 7429-90-5(Al)	20–35% (20–40%) *
Nickel	7440-02-0(Ni)	3–10% (3–10%) *
Iron	7439-89-6(Fe)	10–25% (15–40%) *
Potassium Hydroxide Sodium Hydroxide Lithium Hydroxide	1310-58-3 1310-73-2 1310-65-2	0–15% (0–15%) *

* updated information (2025).

**Table 3:** Section from the X-ray diffraction index sheet for the compound Ni(OH)_2_ identified by the first three most important maxima (I%) recorded (PDF 00-014-0117).

2θ	D (Å)	I	h	k	l
19.258	4.605	100	0	0	1
33.064	2.707	45	1	0	0
38.541	2.334	100	1	0	1
39.098	2.302	2	0	0	2
52.100	1.754	35	1	0	2

## Data Availability

The original contributions presented in this study are included in the article. Further inquiries can be directed to the corresponding authors.
